# Cultural immersion in the education of healthcare professionals: a systematic review

**DOI:** 10.3352/jeehp.2019.16.4

**Published:** 2019-01-31

**Authors:** Marty Jacob Brock, Levi Bryant Fowler, Johnathan Gill Freeman, Devan Cord Richardson, Lisa Jayroe Barnes

**Affiliations:** Department of Physical Therapy, School of Health Related Professions, University of Mississippi Medical Center, Jackson, MS, USA; Hallym University, Korea

**Keywords:** Cultural immersion, Education, Healthcare

## Abstract

**Purpose:**

With the ever-changing cultural makeup of society, the ability to deliver culturally appropriate healthcare is essential. An educational method aimed at increasing cultural knowledge and sensitivity in the education of healthcare professionals is cultural immersion, which creates opportunities for transformational learning through direct interactions with culturally diverse populations. The purpose of this systematic review was to examine the qualitative effects of cultural immersion experiences on graduate-level healthcare professional students.

**Methods:**

A search of the CINAHL (Cumulative Index to Nursing and Allied Health Literature) and ERIC (Education Resources Information Center) databases was performed, utilizing search terms including cultural immersion, cultural sensitivity, educational outcomes, and healthcare professionals. The search was limited to publications within the last 10 years. The articles were screened according to title, abstract, and full-text following the application of inclusion/exclusion criteria. Themes identified within each article were collected and categorized, using a qualitative methodology, into 5 overarching domains to assess the educational experiences. Studies were scored for quality using the qualitative portion of the McGill Mixed Methods Appraisal Tool–2011.

**Results:**

Nine studies incorporating a total of 94 participants with experiences in 14 culturally diverse environments revealing 47 individually identified themes were included in the review. The results indicated that all cultural immersion experiences stimulated increased cultural awareness and sensitivity.

**Conclusion:**

Cultural immersion experiences produced a positive, multi-domain effect on cultural learning in students of the health professions. The results of this review provide support for implementing cultural immersion experiences into the education of healthcare professionals with the goal of increasing cultural sensitivity.

## Introduction

With the ever-changing cultural makeup of society, healthcare professionals can expect to treat an array of different patient populations. The United States Census Bureau identifies the U.S. population as being made up of those who identify as Black, White, Native American, Asian, Pacific Islander, Hispanic or Latino, and 2 or more races. The Bureau also explains that immigrants to the United States originate from Europe, Asia, Africa, Oceania, Latin America, and North America, and therefore bring with them the cultural variations inherent in their homelands. It is not unusual for healthcare professionals in the United States to treat clients whose cultural identities are grounded in a myriad of belief systems, customs, and traditions from countries around the world. This can make the delivery of culturally appropriate healthcare challenging. In order to meet this challenge, healthcare professionals need skills that enable them to provide competent care using culturally sensitive communication, regardless of any cultural differences that may exist [1,2]. Development of these skills during the education process could lead to more culturally competent care.

Healthcare has embraced the emerging reality of an increasingly diverse patient population by requiring some form of cultural competency training in health professional schools. In addition to modifications in academic curricula, studies have explored the effects of cultural competency training among practicing healthcare professionals [3-6]. According to the results of those studies, cultural competency training can improve skills, knowledge, and attitudes among healthcare professionals, leading to improved patient satisfaction and outcomes [3-6]. Educational experiences directed specifically at the development of cultural competency among students of the health professions are an integral part of professional education.

Cultural immersion is an educational method that aims to increase cultural knowledge and sensitivity. The purpose of this type of educational experience is to create opportunities for transformational learning through direct interactions with culturally diverse populations. The goal of this systematic review was to determine the effects of cultural immersion on the education of graduate-level healthcare professionals.

## Methods

The Preferred Reporting Items for Systematic Reviews and Meta-Analyses (PRISMA) guideline for the written presentation of systematic reviews was used to prepare this review. PRISMA is a 27-item checklist for transparent reporting of essential items [7].

### Eligibility criteria

To be included in this review, studies must have evaluated the incorporation of a cultural immersion experience by graduate-level students of the health professions with no prior cultural immersion experience. The results of the studies had to be reported via qualitative methodology, with a pre- and post-experience design. All studies were published in a peer-reviewed format with full-text availability in English. Studies were excluded if they did not include graduate-level students or if the participants’ details were not clearly defined.

### Information sources/search

A search of the CINAHL (Cumulative Index to Nursing and Allied Health Literature) and ERIC (Education Resources Information Center) databases was performed on September 13, 2018 utilizing search terms related to cultural immersion, cultural sensitivity, educational outcomes, and healthcare professionals. The search was limited to publications from January 1, 2007 to September 13, 2018, English-language and peer-reviewed articles, and Boolean phrase results.

### Study selection

The titles were screened by 2 authors, with a third acting to break any ties. Using the resultant articles, abstracts were screened using the inclusion criteria via the same methodology. To determine inclusion, a full-text review of the remaining articles was performed by 2 authors, with a third acting as the tie-breaker. During this step, any duplicates were accounted for and removed from the final total. Table 1 presents a summative analysis of the studies included.

### Data collection process and data items

The selected articles were analyzed by the authors in a round-table discussion during 2 sessions on consecutive days. Prior to the sessions, each author reviewed the articles that would be the focus of that day’s discussion. The data extracted from the articles consisted of diverse themes identified within the articles from various forms of qualitative information, such as journal entries, focus groups, narrative reports, and interviews. A third session was conducted to categorize the themes into similar groups; this discussion resulted in a classification of the following overarching domains: cognitive, affective, perceptual, cultural dissonance, and skills/engagement.

In order to consistently summarize the themes, a consensus regarding definitions was required. The cognitive domain was defined as learning related to conscious intellectual activities such as thinking, reasoning, or knowledge acquisition [8]. The affective domain was defined as learning involving feelings or emotions [8]. The perceptual domain was defined as learning that influenced the participant’s awareness of his or her surroundings through senses and/or spirituality [8]. Cultural dissonance was defined as a lack of agreement with the culture in which the participant was immersed [8]. The skills/engagement domain was defined as engagement with a culturally different native population and/or skills gained through those engagements [9].

### Risk of bias assessment

Studies were scored for quality using the qualitative portion (section 1) of the McGill Mixed Methods Appraisal Tool–2011 (MMAT) [10]. The MMAT is an instrument constructed to appraise the quality of qualitative studies, with a scoring procedure outlined in section 1 of the instrument. The qualitative portion of the MMAT assesses each article in 4 categories, resulting in a percentage score of quality. The categories include an assessment of the sources used within each article, the process used to analyze the data, consideration of how the results related to the context, and consideration of how the results related to the researcher’s influence. The authors discussed the quality criteria and applied them to each of the articles to obtain a quality score.

## Results

### Study selection

**Fig. 1.**
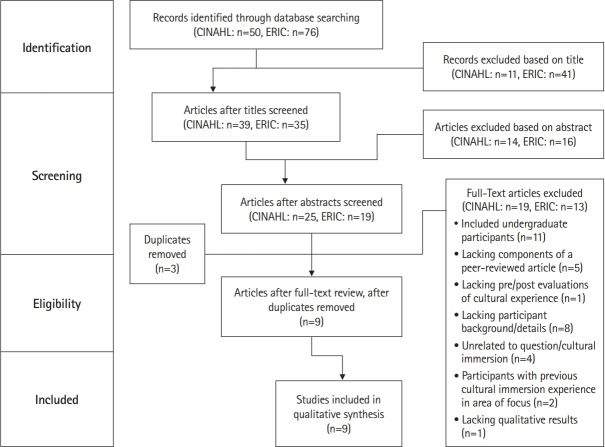
Study selection process using the Preferred Reporting Items for Systematic Reviews and Meta-Analyses (PRISMA) checklist. CINAHL, Cumulative Index to Nursing and Allied Health Literature; ERIC, Education Resources Information Center.

The study selection process is presented in Fig. 1. A total of 126 articles were identified through the electronic search. After the title screen, 74 articles remained. Following review of the abstracts, 30 articles were eliminated, leaving 44 for review. Three of these were removed due to duplication within the databases, leaving 41 articles for consideration using the inclusion/exclusion criteria. After full-text review, 9 studies were selected for inclusion.

### Study characteristics

The articles included in this review incorporated a total of 94 participants with experiences in 14 different culturally diverse environments across 9 countries (Table 1). The individualized outcomes of the studies revealed 47 unique themes. These themes were analyzed and organized into 5 overarching domains that were identified by the researchers through qualitative methodology.

Table 2 outlines how the themes fit into the overarching domains. The results from 2 of the articles showed growth among the participants in all 5 of the domains, and 2 other articles showed participant growth in 4 of the domains. Three of the articles demonstrated participant growth in 3 of the domains, and 2 others showed participant growth in 2 of the domains. The most prevalent type of growth seen among the participants was related to the perceptual domain, with 15 of the original individual themes falling under this designation. Six of the original individual themes were categorized into the skills/engagement domain, indicating that this was least prevalent learning experience as a result of immersion.

### Risk of bias

The results from the MMAT are presented in Table 3. Four articles received a score of 100% on the MMAT, indicating a low risk of bias. Five articles received a score of 75%, indicating that the articles did not meet 1 of the criteria within the assessment tool. The mean score was 86.11%, signifying a high overall quality score.

### Five overarching domains

Seven of the 9 articles demonstrated participant growth in the cognitive domain [8,9,11-15]. In those studies, the authors identified 9 emerging themes related to learning and growing at a cognitive level based on the participants’ reflections. Some of the participants explained that they experienced cognitive growth through improved knowledge, understanding, and realization of cultural differences [8,9,11,12,14]. Others expressed an increase in consciousness and self-awareness leading to a desire to learn more about cultural differences [11-13,15]. In addition to the purely cognitive act of learning, participants expressed that increased knowledge and understanding occurred through interacting and building connections with the people and communities in which they worked [8,9,14].

Five articles demonstrated participant growth in the affective domain [8,13-16]. Within these studies, 9 emerging themes reflected affective learning. A broad spectrum of emotional growth took place among the participants. Some expressed feeling a greater sense of appreciation and gratitude for their own standard of living [13-16]. Many reported gaining a renewed passion and sense of purpose in their field of study [13-15]. Not all learning was associated with positive emotions, with some participants expressing mixtures of anger, sadness, and shame [8,14,15].

All 9 of the articles demonstrated growth in the perceptual domain [8,9,11-17]. After having been outsiders in a foreign country or culture, participants reported having a newfound ability to examine the validity of intrinsic values, socio-political issues, and privileges of their own societies [9,11-13,15,16]. They became more aware of negative issues such as poverty, sub-standard living environments, racial segregation, discrimination, and inequity [11,13,14]. Participants described an increased sense of connection among body, spirit, mind, and the world around them [8,9,15-17]. The immersion experience allowed students to discern an increased ability to approach others different from themselves [16]. Increased perceptiveness led to increased openness, appreciation of basic necessities, and decreased prejudice [12,14,17]. Students reported a heightened awareness of preconceived assumptions, beliefs, unprocessed feelings, and insecurities [9,11,15,16]. Through observation of unfamiliar customs, participants reported an improved outlook on the importance and value of cultural differences [16].

Five articles revealed growth in cultural dissonance [8,12,14,15,17]. Eight emerging themes were related to disagreements with the cultures in which the participants were immersed. Participants reported frustration with the constraints of western medicine related to its negative view towards alternative medicine [17]. Some participants experienced feelings of apprehension due to language barriers, unsafe drinking water, and obstacles such as ethnocentricity [12,14,15]. Cultural dissonance was shown in the immersion experiences through displays of stereotyping, judgment of others, and avoidance of reflecting on issues of diversity [8]. As part of their experiences of a foreign environment, participants described negative psychological or behavioral changes, such as difficulty coping, insomnia, uncertainty, anticipation, fatigue, and even physical illness [12,15].

Five articles discussed growth in the skills/engagement domain, with 6 emerging themes [9,13-15,17]. Some participants reported connecting with the local populations through engagement with the people and their customs [19,13-15]. Students reported an emergent desire to incorporate some of the local practices into their daily lives [9,14,17]. Some participants reported sensing an internal change resulting from personal engagement with the population in which they were immersed. This type of reflective self-assessment could benefit future healthcare endeavors [9,14,15].

## Discussion

Our findings suggest that cultural immersion experiences can produce a positive multi-domain effect on learning in students of the health professions. In each study, learning occurred through placing individuals in culturally unique situations that provided a broad array of learning opportunities.

The cognitive domain illustrated participants’ ability to acquire knowledge while participating in immersion experiences. The affective domain reflected participants’ emotional growth, including both positive and negative emotions. The perceptual domain resulted in the greatest amount of growth across the studies. Participants reported improved awareness of how to interact with cultures different from their own, and an improved outlook when considering the importance of cultural differences. The cultural dissonance domain illustrated the frustrations participants felt when comparing the culture they were immersed in to their own. The skills and engagement domain illustrated gaining new skills and the ability to connect with varied populations.

Similar findings have been reported in studies by Larsen and Reif [18], Tomlinson-Clarke and Clarke [19], and Charles et al. [20], all of whom reported that improved cultural awareness and sensitivity followed cultural immersion experiences. A systematic review conducted by Clifford et al. [21] reported an increase in cultural competency through knowledge, attitudes, and awareness, indicating growth in the cognitive, affective, and perceptual domains following immersion experiences. Work by Tremethick and Smit [22] and Conroy and Taggart [23] reported the importance of cultural immersion in the preparation of culturally competent healthcare professionals for the development of clinical and interpersonal skills, suggesting that immersion experiences may lead to improved patient care.

Future studies of cultural immersion experiences may benefit from the inclusion of a wider variety of healthcare professions. Six of the 9 studies included herein were in the counseling field, with limited information available for other healthcare professions such as physical therapy, occupational therapy, speech therapy, medicine, and nursing.

Some limitations of this study should be acknowledged. The review was limited to studies in English, which could have omitted pertinent studies. Additionally, studies were excluded if the subject population characteristics were not clearly defined, which could have resulted in otherwise acceptable studies being omitted due to a lack of information.

Due to the qualitative nature of this research, there is a risk of potential bias. Bias could have occurred as a result of the original authors assigning themes to the data they collected. The studies varied in the intensity of the cultural immersion experiences. Some participants were placed in cultures that were only slightly different from their own and others were immersed in dramatically different cultures. This limits the ability to compare outcomes across individual studies. Bias could also have occurred when the themes were assigned to the 5 overarching domains identified within this systematic review.

In conclusion, this review showed that the incorporation of cultural immersion in the education of students of the health professions had an overall positive effect on improving cultural awareness and sensitivity. Participants’ reflections revealed widespread growth across multiple domains. By implementing this proven method of stimulating growth across multiple learning domains, healthcare educators can reasonably expect students to attain a higher level of cultural sensitivity, with the potential of elevating the quality of healthcare in multicultural environments.

## Figures and Tables

**Table 1.** Summary of articles included in this review

Study	Quality score (McGill score, %)^a)^	Healthcare field	Study participants	Setting	Intervention/assessment	Outcomes/themes
Choi et al. [13] (2015)	100	Counseling	Five master’s-level counseling students (4 females, 1 male) attending a midsized university located in the United States. All participants were White Americans, ages 26 to 38.	South Africa	Participants were involved in a 14-day study abroad class in South Africa. A variety of educational, cultural, and field experiences were undertaken by the participants. Viewpoints about the cultural immersion experience were gathered through semi-structured interviews 6 months after the experience. A qualitative methodology was used to analyze information gathered during 60- to 90-minute interviews. Data analysis revealed 5 major themes.	5 Themes: (1) the meaning of being American; (2) socio-political awareness; (3) engagement with South Africans and their local communities; (4) appreciation of life; and (5) commitment to change
Crowe et al. [17] (2016)	75	Occupational therapy	Thirty-six students from the United States, ages 20–60+ participated in an immersion experience.	Mexico	An academic course involving a 12 day experience in Oaxaca, Mexico immersed participants in a foreign culture. Using qualitative analysis with guided questions and focus groups, 4 main themes were derived from participants’ opinions and thoughts about the experiences.	4 Themes: (1) natural remedies; (2) mind, body, spirit, connection; (3) increased openness; and (4) challenges of integrating traditional and western medicine
Goodman [14] (2016)	75	Nursing	Seventeen graduate nursing students, ages 24–64 participated in an immersion experience.	Guatemala	Students kept reflective journals of their experiences including teaching, healthcare, and general cultural interaction with people in rural villages. Notes and journals were collected from participants and analyzed to reveal 9 themes.	9 Themes: connection with: (1) community; (2) environment; (3) local partners; (4) one’s own level of comfort; (5) others (patients, colleagues, classmates); (6) self and internal peace; (7) the future; (8) other underserved communities; and (9) nursing profession
Hipolito-Delgado et al. [9] (2011)	75	Counseling	Three first-year female graduate students at an urban university in the United States participated in the experience. Two identified as heterosexual and 1 identified as homosexual.	Students were allowed to choose a setting with a culturally different population.	Participants were enrolled in a semester-long assignment in an academic course in which they selected a cultural environment different from their own based on one or more of the following: race, ethnicity, gender, sexual orientation, ability, or age. Students were immersed in the environment, kept weekly journals, and compared those observations to the counseling literature related to their community of choice.	3 Themes: (1) increased awareness; (2) increased knowledge; and (3) increased skills
Ishii et al. [8] (2009)	100	Counseling	Fifteen female master’s-level counseling students, ages 24–56, from a university in the United States participated (11 European Americans, 3 African American, and 1 bi-ethnic).	New Mexico	Students participated in 3 preparatory meetings with various educational methods prior to the experience. Participants travelled to cultural and historical sites in New Mexico, USA, representing Aztec, Hispanic, and Pueblo Native American cultures. Follow-up data resulted in 5 emergent themes.	5 Themes: (1) cognitive reactions; (2) affective reactions; (3) perceptual reactions; (4) empathy; and (5) cultural dissonance
Peiying et al. [12] (2012)	75	Occupational therapy, physical therapy speech pathology	15 Female participants from Australia, ages 22–30 participated in the immersion experience.	China, India	Students participated in a 4-week immersion experience in either China or India. Journals and guiding questions were completed throughout the experience. Narrative data were analyzed by blinded researchers who classified feedback into 5 major themes.	5 Themes: (1) increased vigilance and adaptation to environment; (2) uncertainty and anticipation; (3) grappling with supremacy; (4) recognizing and appreciating differences; and (5) cultural immersion and development
Prosek and Michel [16] (2016)	100	Counseling	13 Master’s level students, ages 21–37, from programs across the United States participated (12 females, 1 male, 10 European American, 1 African American, 1 Hispanic, and 1 multiracial).	Ireland	The counseling students participated in a 13-week multicultural/diversity course. The course involved 11 weeks of education on Irish culture followed by a 10 day cultural immersion experience in Ireland. Students submitted reflection papers and completed interviews used for data analysis revealing 3 themes.	3 Themes: (1) cultural self-awareness; (2) witnessing peer growth; and (3) global connection
Shannonhouse et al. [15] (2015)	100	Counseling	10 Graduate students, ages 23–32, participated. (9 females, 1 male, 9 European American, 1 Asian American).	Costa Rica	The students participated in a 3-week cultural immersion experience course in Costa Rica. They submitted 210 structured journals that were analyzed using qualitative methodology, revealing 9 themes.	9 Themes: (1) personal characteristics; (2) past experiences; (3) coping; (4) emotional reactions; (5) communications; (6) relational connections; (7) encouragers/barriers; (8) personal and professional changes; and (9) awareness
Smith-Augustine et al. [11] (2014)	75	Counseling	Five African American female graduate students, ages 20–25, from a university in the United States participated.	Belize	The students participated in a 3-week study abroad in Belize where they interacted with persons from diverse cultural backgrounds. Participants submitted journals for analysis, which revealed 4 themes related to the cultural immersion experience.	4 Themes: (1) discrimination and prejudice; (2) cultural pride and appreciation; (3) cultural sensitivity; and (4) self-awareness

a) Scoring based on McGill Qualitative Methodological Quality Criteria. See Table 3 for scoring details.

**Table 2.** Placement of individual themes into overarching domains

Study	No. of themes	Cognitive	Affective	Perceptual	Cultural dissonance	Skills/engagement
Choi et al. [13] (2015)	5	Commitment to change	Appreciation of life	Meaning of being an American, socio-political awareness		Engagement with South Africans and their local communities
Crowe et al. [17] (2016)	4			Mind, body, spirit connection, increased openness	Challenges of integrating traditional and western medicine	Natural remedies
Goodman [14] (2016)	9	Connection with: community, others, the future	Connection with: self and internal peace, nursing	Connection with: environment	Connection with: one’s own comfort level	Connection with: local partners, underserved communities
Hipolito-Delgado et al. [9] (2011)	3	Increased knowledge		Increased awareness		Increased skills
Ishii et al. [8] (2009)	5	Cognitive reactions	Affective reactions, empathy	Perceptual reactions	Cultural dissonance	
Peiying et al. [12] (2012)	5	Cultural immersion and development		Recognizing and appreciating differences	Increased vigilance and adaptation, uncertainty and anticipation, grappling with supremacy	
Prosek and Michel [16] (2016)	3		Global connection	Cultural self-awareness, witnessing peer growth		
Shannonhouse et al. [15] (2015)	9	Encouragers/ barriers	Personal characteristics, emotional reaction, relational connections	Past experiences, awareness	Coping, communications	Personal and professional changes
Smith-Augustine et al. [11] (2014)	4	Cultural pride and appreciation		Discrimination and prejudice, cultural sensitivity, self-awareness		
Themes within domain		9	9	15	8	6

Cognitive: learning related to conscious intellectual activities such as thinking, reasoning, or knowledge acquisition. Affective: learning involving feelings or emotions. Perceptual: learning that influenced the participant’s awareness of his or her surroundings through senses and/or spirituality. Cultural dissonance: lack of agreement with the culture in which the participant was immersed. Skills/engagement: engagement with a culturally different native population and/or skills gained.

**Table 3.** McGill Qualitative Methodological Quality Criteria

Study	1.1. Source relevance	1.2. Process relevance	1.3. Content relevance	1.4. Researcher influence	Scoring (%)
Choi et al. [13] (2015)	Yes	Yes	Yes	Yes	100
Crowe et al. [17] (2016)	Yes	Yes	Yes	No	75
Goodman [14] (2016)	Yes	Yes	Yes	No	75
Hipolito-Delgado et al. [9] (2011)	No	Yes	Yes	Yes	75
Ishii et al. [8] (2009)	Yes	Yes	Yes	Yes	100
Peiying et al. [12] (2012)	Yes	Yes	Yes	No	75
Prosek and Michel [16] (2016)	Yes	Yes	Yes	Yes	100
Shannonhouse et al. [15] (2015)	Yes	Yes	Yes	Yes	100
Smith-Augustine et al. [11] (2014)	No	Yes	Yes	Yes	75

## Supplementary materials

Supplement 1. Audio recording of the abstract.
